# Evaluating *Now I Know* mHealth intervention promoting HPV vaccine completion among young women attending federally supported clinics

**DOI:** 10.1007/s10552-025-02046-8

**Published:** 2025-10-11

**Authors:** Su Kyung Kim, Rebecca Duncan, Jesse Chittams, Anne M. Teitelman

**Affiliations:** 1 College of Nursing, Thomas Jefferson University, Philadelphia, PA USA; 2 School of Nursing, University of Pittsburgh, Pittsburgh, PA USA; 3 School of Nursing, University of Pennsylvania, Philadelphia, PA USA

**Keywords:** Primary prevention, Cancer, Young women, HPV vaccine, Intervention

## Abstract

**Purpose:**

This study evaluated the feasibility, acceptability, usability, and HPV vaccine completion rate of the *Now I Know* (NIK) mHealth intervention to promote HPV vaccine completion among minority and low-income young women.

**Methods:**

This quasi-experimental pilot study recruited 35 women aged 18–26 at two federally supported outpatient clinics in a large city in the Northeastern United States. Participants were allocated to two groups: 24 received the NIK intervention plus usual care and 11 received usual care only. After completing a baseline assessment, intervention group participants launched the NIK mobile app, which entailed HPV vaccine education, self-managed vaccine record & reminder, and Q&A features. Follow-up visits were conducted at 2 and 6 months. Feasibility was assessed through screening, recruitment, fidelity, and retention. Acceptability and usability were evaluated via survey and exit interview. The primary outcome—HPV vaccine 3-dose series completion—was analyzed using descriptive statistics.

**Results:**

This study demonstrated feasibility by reaching the recruitment target (n = 35) and high retention rate (89%). Participants reported the app was acceptable, appreciating reliable information, convenient access, providing a personal touch, and raising awareness. Regarding usability, users found the app easy to navigate, accessible, well organized, and user-friendly. The HPV vaccine completion rate was higher in the intervention group (55%) than in the usual care group (45.5%).

**Conclusion:**

Findings showed improved vaccination completion among those using the Now I Know mHealth intervention (compared to usual care), acceptability of the intervention, and feasibility of recruiting, retaining, and delivering the intervention to promote HPV vaccine completion among young women in federally subsidized healthcare settings.

## Introduction

Annually, more than 37,000 people in the U.S. develop HPV-associated cancers, resulting in 6,482 deaths and costing an estimated $9.36 billion [1, 2]. Cervical cancer, the most common HPV-associated cancer among women in the U.S., is associated with approximately 4,000 deaths per year [3]. However, 92% of these cancers are preventable with HPV vaccine series completion. While the Centers for Disease Control and Prevention (CDC) advises a 2-dose HPV vaccine before age 15 and a 3-dose series for ages 15–26 [4, 5], HPV vaccination completion rates are particularly low among young adult women aged 18–26, compared to those in the younger age group [6, 7]. National Health Interview Survey Reports from 2018 indicate that while 53.6% of women aged 18–26 initiated HPV vaccines, only 35.3% completed the HPV vaccine series [7], well below the 80% Healthy People 2030 target. A health system-based survey (n = 265,554) reported a 42.2% HPV vaccine initiation rate and 33.6% completion rate among women aged 18–26. Disparities persist among young women, with lower completion rates among women from lower-income backgrounds and racial and ethnic minority groups, compared to their higher-income and/or white counterparts [8, 9]. Rural women had a lower HPV vaccine completion rate compared to urban women, even when the vaccine was free [10]. Black and Hispanic women are less likely to complete compared to white women [8, 9]. Women with private insurance are more likely to complete the series than those with Medicaid or other types of insurance [8]. Considering Black women exhibit the highest incidence of HPV-associated cancers, disproportionate completion rates could exacerbate cancer disparities [11, 12], indicating the unmet needs of HPV vaccination intervention targeting low-income young adult women of color.

Identified barriers to HPV vaccine completion include cost, inconvenience of returning for additional doses, lack of awareness of additional doses, and forgetting to return to care [8, 13]. While randomized controlled trials (RCTs) have shown targeted interventions increase HPV vaccine uptake in children [14–17], few studies have focused on economically disadvantaged young adult women [16, 18]. Since women aged 18–26 begin to make their own health decisions, interventions targeting children and their parents are not directly applicable to this population. Few studies have tested HPV vaccine completion interventions targeting young adults aged 18–26, and most of these primarily recruited from university student health settings [19–22]. HPV vaccine completion rates among young adult women attending federally supported/qualified health centers are low (20%)[23]. Women attending federally supported health clinics often differ from college women with respect to socio-economic characteristics such as race/ethnicity, insurance, and education—all factors associated with HPV vaccine disparities. Women attending federally supported clinics reported age-related misunderstandings and a lack of communication with providers as HPV vaccination barriers [24].

By aligning with young people’s mobility, enabling individuals to self-manage vaccine records, and ensuring consistent access to care, mobile Health (mHealth) offers a promising approach to promoting HPV vaccine completion among this group [25]. mHealth can effectively bridge gaps in traditional provider-driven record-keeping, especially for those who struggle with in-person healthcare access in rural or underserved areas. Distributing health information via mobile phones facilitates the delivery of targeted education and reminders directly to target users.

Systematic reviews of HPV vaccination interventions highlight mHealth’s potential to improve uptake [24] and decision-making [26–29]. Mobile applications (apps) featuring targeted educational content with reminders have proven more effective compared to generic reminder messages [30]. mHealth is particularly effective between clinic visits, providing trustworthy, accessible vaccine information that can reduce reliance on less reliable online sources. For example, a 2016 mHealth study noted that a 7-day text message intervention significantly increased HPV vaccine knowledge and intent among Korean American young women aged 21–29, with 30% vaccine initiation [31]. Similarly, a 2020 study indicated that mHealth effectively promoted HPV vaccination among men who have sex with men [32]. Despite mHealth’s potential, economically disadvantaged and minority young adult women, who frequently utilize reproductive healthcare services, remain underrepresented in mHealth HPV vaccination studies. Thus, the “*Now I Know”(NIK)* mHealth intervention was designed to improve health literacy along with features for self-managed record-keeping and reminders [33]. This pilot study aimed to (1) describe whether participants assigned to the NIK intervention group had higher HPV vaccine completion compared to those in the usual care group, (2) assess the acceptability of the intervention to the target population, and (3) evaluate the feasibility of delivering content, recruiting, and retaining participants. The primary outcome was descriptive, focusing on HPV vaccine completion, with the hypothesis that the NIK group would demonstrate a higher rate than the usual care group.

## Methods

### Design

This study focused on HPV vaccine completion, defined as following the initial dose with 2- and 6-month doses, often involving returning for a vaccine-only appointment between routine visits. Completing the series required awareness of the need for additional HPV vaccines and support to return for follow-up visits twice over 6 months at specific intervals. Consistent with the social-cognitive factors that influence HPV vaccine completion, we designed the NIK mHealth intervention [33] to reach the target population outside the clinic setting as an easily accessible and supportive resource.

This study was nested within a larger study that previously assessed HPV vaccine initiation, defined as receiving the first dose of the HPV vaccine during a routine visit [34]. Participants in the larger study were aged 18–26, attended federally supported clinics, had not received the HPV vaccine, had vaginal sex, were able to complete the study visits, agreed to receive reminder texts or e-mails, and demonstrated English comprehension at or above a fifth level. Among participants in that study, 35/60 initiated the HPV vaccine. All who initiated the HPV vaccine were invited to participate in the NIK mHealth intervention study, focused on completing the HPV vaccine series. All approached participants enrolled in the NIK mHealth intervention study following the consent process.

We used a pilot quasi-experimental design with some participants receiving usual care only and others receiving usual care plus the NIK app. Eligible participants completed the informed consent process and were assigned to two groups: the NIK group and the comparison group. The university’s institutional review board approved the study.

### Theoretical framework

Formative research exploring underlying beliefs influencing HPV vaccine completion behavior among young, low-income women was used to develop educational content included in the mHealth intervention [35]. The assessment of these beliefs was guided by the Integrated Behavior Model, which posits that behavioral changes are driven by underlying beliefs [36]. Based on the beliefs identified [35], we developed targeted content to enhance knowledge and target key beliefs to favorably influence HPV vaccine completion behavior.

Key beliefs included understanding that completing the HPV vaccine series reduces the risk of HPV infection and cervical cancer. Beliefs influencing control (self-efficacy) focused on overcoming personal challenges associated with completion, such as remembering to schedule appointments, overcoming time constraints, and transportation challenges. Finally, beliefs influencing social norms revolved around family, partners, and friends approving or supporting HPV vaccination. To advance HPV health literacy, we also included relevant information, e.g., “You need to receive a series of 3 vaccines to get the full benefit of the HPV vaccine.”

App development was guided by the Technology Acceptance Model (TAM), which suggests that perceived usefulness and perceived ease of use are the strongest predictors of whether a technology will be accepted and adopted [37]. The development of the app was also guided by the User-Centered Design process [37, 38]. Throughout the development process, we involved end-users to ensure acceptability and usability, thereby increasing the likelihood of use of the app and promote completion of the HPV vaccine among our target population.

### Intervention

The NIK mHealth intervention was developed to promote HPV vaccine series completion among young adult low-income women (including those who attend federally supported clinics). Functional components of the app included enhancing health literacy through engaging educational content, providing a personalized HPV vaccine administration record, and incorporating an appointment reminder system aligned with the recommended HPV vaccination schedule. To ensure the intervention was culturally relevant and user-friendly, a Community Advisory Board (CAB) was convened to inform app development guided by the TAM and User-Centered Design framework. Monthly meetings were held to facilitate ongoing collaboration, and iterative feedback was systematically integrated into app design. Feedback encompassed various areas of the user experience, including visual design, language clarity, and culturally relevant content. The final NIK mHealth smartphone app was downloadable for iOS (Apple) and Android platforms to study participants at no cost.

Health literacy education and motivational content was delivered via a rotating series of 54 stories, highlighting two stories each week through push notifications. The stories were specifically crafted to portray young adult women encountering challenges similar to those faced by the target population in completing HPV vaccination. Additionally, each unique story addressed one or more salient beliefs identified in the initial formative research and provided health literacy information on HPV, cervical cancer, the HPV vaccine, and other prevention strategies such as Pap tests and condom use. The app also offered individualized profiles for users, enabling recording of HPV immunization events and creating reminders through push notifications.

Launching the app, users encountered several tabs at the top providing access to distinct features within the application, for example, “Stories.” The “Vaccination Status” tab allowed users to view the date of their upcoming scheduled HPV vaccine and track self-managed HPV vaccine records. Appointment reminder push notifications were sent out 1 week, 3 days, and 1 day prior to their second and third dose appointments. The “Q&A” forum provided an opportunity for users to post questions and receive responses from other users and our team of nurses/nurse practitioners to provide accurate information. We provided guidelines for posting in the app, and the Q&A forum was closely monitored by study staff for any offensive or inaccurate content. A detailed description of the NIK app and the development process has been described elsewhere [33].

### Setting

The study setting comprised two outpatient clinics in a large, diverse metropolitan area in the Northeastern United States. The first clinic operated as a federally supported family planning program within an OB/GYN practice. The second was a federally qualified health center (FQHC) that provided family planning services for adolescent patients up to 26 years old. Both clinics offered subsidized care for low-income patients, and HPV vaccines were offered at no cost as part of this study.

### Allocation to intervention

The first 11 recruited participants comprised the comparison group. This group received usual care, which involved standard vaccine education and routine appointment reminders for HPV vaccine follow-up doses generated from the clinic site. The usual care group was informed about the three-dose HPV vaccine schedule, scheduled for the follow-up vaccine appointments, and received reminders of their vaccine appointments through an automated phone message. The subsequent 24 recruited participants were allocated to the intervention group and received the NIK app in addition to usual care. Since the NIK app was a new intervention, we allocated more participants to the intervention arm of the study as we sought to obtain a greater density of information from that group.

### Participants

Thirty-five female participants aged 18–26 who had just received the first dose of the HPV vaccine were recruited to participate in the study with convenient sampling. Inclusion criteria consisted of having no contraindications for subsequent vaccine doses (based on initial vaccination, e.g., no untoward reaction), a smartphone capable of downloading the NIK app, and the ability to complete surveys in English. Study exclusion criteria included pregnancy. As compensation, participants received a 35 USD gift card at the baseline, 2- and 6-month follow-up study visits.

### Data collection

We assessed feasibility through screening, recruitment, treatment fidelity, and retention rates. HPV vaccination rates and intervention acceptability were assessed through surveys (Table 1. Measures and Outcome). Participants completed confidential surveys using a computer-assisted self-interview (CASI) at three points: initially before the intervention (baseline), after the second vaccination (2 months), and after the last vaccination (6 months). For participants enrolled in the intervention group, study staff introduced participants to the NIK app upon completing the baseline survey and guided them as they launched the app on their phones. We conducted the 2-month follow-up visit approximately 2 months after enrollment, and the 6-month follow-up visit approximately 6 months after enrollment, each with a 2-week grace period. Phone call and/or text reminders were sent beginning two weeks prior to the follow-up study visit dates. Follow-up visits were conducted in person either at the clinic or at the university office, regardless of whether the second or third dose had been received.

**Table 1:** Measures & outcome table

Outcomes	Measures	Results
Feasibility		
Screening	# Meeting inclusion criteria	*n* = 35
Recruitment	% Consenting	100% (*n* = 35)
Treatment fidelity	% mHealth delivered (downloaded)	100% (*n* = 35)
Retention	% Completing follow-up	100% at 2 month (*n* = 35)
		88.6% at 6 month (*n* = 31)
Outcome (HPV vaccine completion rates)	% HPV vaccine completion	2nd HPV vaccine
		App group 79.2% Comparison group 63.6%
		3rd HPV vaccine
		App group 55.5% Comparison group 45.5%
Acceptability	Acceptability Survey (5-point Likert scale)	See acceptability survey result table
	Acceptability feedback	See qualitative feedback table
Usability	Usability feedback	See qualitative feedback table

The exit interview was conducted in person after the completion of the 6-month survey, for NIK app participants only, since the goal was to gather more detailed information about the acceptability and usability of the NIK app. The data collector provided the participant with a printed version of the questions. The data collector then read each question to the participant and typed their responses verbatim into the computer database. The data collectors were students in the healthcare professions (nursing, public health, medicine). Training consisted of becoming familiar with the questions, practicing in reading the questions out loud, and typing responses exactly as the participant had stated. The exit interview usually lasted 10–15 min. Data collectors entered the responses verbatim directly into the study database during interviews.

### Measures

#### Participant characteristics

Participant demographic and health variables evaluated included age, race, ethnicity, mother’s education, recent well-woman’s exam, history of sexually transmitted infection, condom use in the past two months, HPV-related knowledge, and intention to complete HPV vaccine series. HPV intention to complete HPV vaccine series were measured in 5-point Likert agree/disagree questions with response options ranging from strongly disagree, disagree, neither agree nor disagree, agree, and strongly agree. HPV intention 5-point Likert scale was recoded into a binary outcome: “strongly disagree,” “disagree.” and “neither agree nor disagree” were coded as “no,” while “agree” and “strongly agree” were coded as “yes.”

#### Feasibility

The feasibility of conducting the study was assessed by measuring the eligible participants’ willingness to engage in the research. This was determined through achieving the desired sample size and analyzing retention rates. Additionally, feasibility was assessed based on developing and implementing the mHealth intervention and conducting the study in two distinct clinic environments.

#### Acceptability

Participant feedback on the perceived acceptability of the intervention was gathered at the 6-month survey. All study participants were asked four general questions related to adherence-support satisfaction, which assessed participants’ views about the various modalities of assisting with vaccine completion without specifically referencing the app. Questions pertained to getting reminders about the next HPV vaccine appointment, keeping track of HPV vaccine appointments on a calendar, discussing the HPV vaccine with others, and reading about other people’s experiences with the HPV vaccine. Responses to acceptability were measured using a 5-point Likert scale and recoded into a binary outcome: “strongly disagree,” “disagree,” and “neither agree nor disagree” were coded as “no,” while “agree” and “strongly agree” were coded as “yes.” For example, one item assessed: “Getting reminders about my next HPV vaccine is helpful for me to complete the vaccine series,” with a 5-point Likert agree/disagree response option. Participants (in the NIK app group) were also asked one open-ended question about intent to use the app before their next vaccine. At 6 months, data collectors conducted a brief exit interview among NIK participants, soliciting general feedback on the NIK app acceptability and various features, and asking if they would recommend the app to others.

#### Usability

Usability, defined as perceived ease of use, of the NIK app was assessed by evaluating the degree to which users believed using the technology was uncomplicated. This was determined by asking for feedback through two user-experience questions on the 6-month exit interview: what they found easy and what they found hard about using the app (What is easy/hard about using the NIK app?). Usefulness was also assessed on the 6-month exit interview by asking several questions, for example: What did you think about the push notifications? What have you learned or taken away from reading the stories so far? What have you learned from reading the Q&A so far? Which features of the app did you like the most?

#### HPV vaccine completion rate

HPV vaccine completion rate was measured based on participants’ survey responses on the receipt of the second and third vaccines. Receipt of the HPV vaccine was assessed with a single survey question: “Did you receive the relevant (2nd or 3rd) dose of the HPV vaccine (yes/no)?” at each time point and verified through medical records.

### Analysis

#### Quantitative analysis

Descriptive statistics were employed to summarize demographics and outcome variables. To contrast participant characteristics between intervention groups at the outset, Fisher’s exact test was utilized. Frequencies and percentages were reported for categorical variables, and means or medians were used for continuous variables where appropriate. Group-level comparisons (e.g., between intervention and comparison groups) were presented descriptively at baseline, 2 months, and 6 months to illustrate trends in HPV vaccine uptake over time. All analyses were conducted using SPSS version 29 [37].

#### Participant feedback analysis

The responses were typed verbatim into a database by data collectors. Content analysis was used to examine participant responses to acceptability and usability questions in the exit interviews, focusing on perspectives and user experiences. Two coders were involved during the content analysis process. Responses to each specific question were entered into a table. Responses pertaining to each question were analyzed for patterns and grouped together. Each of the two coders then used the compare and contrast content analysis method to identify patterns of common responses. Discrepancies in the coding or analysis were discussed until consensus was reached. Common themes were identified as representing at least 20% of the responses [36].

## Results

### Sample and participant characteristics

**Fig. 1:**
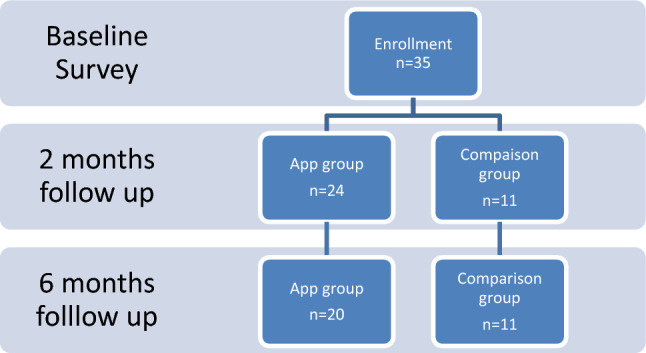
Consort diagram of progress through the trial

Overall, 35 participants enrolled and completed the baseline survey. The 2-month follow-up visit had 100% retention (n = 35), and the 6-month follow-up had 89% retention (n = 31). At 6 months, 4 participants in the intervention group were lost to follow-up (Fig. 1).

In the baseline survey (n = 35), all participants were aged 18–26, with a mean age of 22 years. Most participants were African American/Black (71%), followed by White (17%), Hispanic (9%), and Asian/Pacific Islander (6%). Half of the participants were students (54%), and less than half had completed high school education (46%). Half of the participants were working either part time or full time (54%). More than half (57%) had a well woman’s visit within the past year, and 34% were sexually active in the past 2 months (Table 1). One-third of the participants had a history of STI (34%) and had multiple sexual partners (34%). Most participants (80%) responded that their mother finished high school. Less than half (40%) used condoms during their last vaginal sexual encounter. There were no significant demographic, HPV-related knowledge, or HPV-vaccine-completion-intention differences between the app vs. comparison group at baseline. The 3 response options to the HPV-related knowledge questions were recoded into a binary outcome: “No” and “Don’t know” were coded as “no,” while “Yes” was coded as “yes.” Responses to intention to complete the HPV vaccine series were measured using a 5-point Likert scale and recoded into a binary outcome: “strongly disagree,” “disagree,” and “neither agree nor disagree” were coded as “no,” while “agree” and “strongly agree” were coded as “yes.” (Table 2).

**Table 2:** Participant characteristics at baseline by intervention group (N = 35)

Characteristic	Intervention Group	
	App group *n* = 24 Yes *n* (%)	Comparison group *n* = 11 Yes *n* (%)
Are you a student? Student status	14 (58.3)	5 (45.5)
Did you complete high school education?	13 (54.1)	3 (27.3)
Do you work? Employment status (full time or part time)?	14 (58.3)	5 (45.5)
Have you ever had a History of sexually transmitted infection (STI)?	7 (29.2)	5 (45.5)
Did you have multiple sexual partners in the past two months?	8 (33.3)	4 (36.4)
The last time you had sex, were condoms used at last sexual encounter?	7 (30.4)	7 (63.6)
Did your mother finish high school education?	20 (83.3)	8 (72.7)
Did you have your last recent well women’s health or/annual GYN visit?	13 (54.2)	7 (63.6)
Do you aware that a series of 3 HPV vaccine doses are needed to get the full benefits of the HPV vaccine?	20 (83.3)**	9 (81.8)**
How likely is it that you will get the HPV vaccine? Self-reported likelihood of getting the HPV vaccine	6 (25.0)*	5 (45.5)*

+ Yes was derived from a 3-point Likert scale: "True" was coded as "yes," while "Don't know" and "False" were coded as "no."

*Yes was derived from a 5-point Likert scale and included strongly agree and agree

### Feasibility

#### Recruitment and retention

The predetermined recruitment goal was achieved with a successful enrollment of all 35 participants (who completed the first dose of the HPV vaccine as part of the parent study) and a retention rate of 89% at 6-month follow-up. The study was successfully conducted in two clinical settings that offered subsidized healthcare to low-income patients, a patient population that is less often included in young adult HPV vaccine studies.

#### Intervention delivery

The mHealth app was successfully introduced to all intervention group participants with 100% initial exposure. During the baseline visit, a research staff member assisted participants with downloading the app on their phone, reviewed the various features (HPV vaccine education, self-managed vaccine record & reminder, and Q&A features), and asked the participants to select and read one story. Three participants were unable to download the app during the initial visit due to limited storage or their phones not working properly. In these cases, the research staff member demonstrated the app, as described above, on a separate device. The staff then followed up with these participants by phone within 1–2 weeks to assist and confirm that the app had been successfully downloaded. At the 2-month follow-up visit, the app was reintroduced to participants who had changed their phones and had missed reinstalling the app.

### Acceptability

At the 2-month follow-up (NIK app group only), when asked about intention to use the app before their next vaccine appointment, most NIK participants expressed intention to continue using the NIK app before their next vaccine appointment.

At 6-month follow-up, we assessed the acceptability HPV vaccine completion support in both groups through four survey questions and in the NIK group through the exit interview. Since the acceptability questions pertained to HPV vaccine completion, only participants who received the last dose were asked these questions. Overall, participants in the App group reported higher agreement with the helpfulness of the vaccine support features compared to those in the usual care group. Specifically, 100% of App group participants found reminders helpful, versus 80% in the comparison group. Self-managed vaccination record tracking was rated helpful by 100% of the App group compared to 40% in the comparison group. Similarly, 100% of the App group discussing the HPV vaccine with others through Q&A features was helpful, compared to 60% in the comparison group, also showing a marginal difference. In addition, 100% of the App group reported that reading others’ experiences made it easier to complete the vaccine, compared to 40% of the comparison group. All acceptability items showed higher levels of agreement in the App group compared to the comparison group, though statistical significance was marginal in two items (Table 3).

**Table 3:** Acceptability Survey Results by App group at 6 months

Acceptability Item (N = 16)	App Group Yes n/N (%)*	Comparison Group Yes n/N (%)*
Getting reminders about my next HPV vaccine was helpful for me to complete HPV vaccines	11/11(100%)	4/5(80%)
Keeping track of getting the HPV vaccine on a calendar was helpful for me to complete HPV vaccines	11/11(100%)	2/5(40%)
Discussing the HPV vaccine with others made it easy to complete the vaccines	11/11(100%)	3/5(60%)
Reading about other people’s experience with HPV vaccine made it easy to complete HPV vaccines	11/11(100%)	2/5(40%)

*Responses to acceptability were measured using a 5-point Likert scale and recoded into a binary outcome: “strongly disagree,” “disagree,” and “neither agree nor disagree” were coded as “no,” while “agree” and “strongly agree” were coded as “yes.”

Note: Responses were collected using a 5-point Likert scale and recoded into a binary outcome

At 6-month follow-up, participants shared feedback on the app’s acceptability during the exit interview (Table 4). They highlighted the app’s provision of valuable information, effective reminders, and its role in fostering HPV awareness, describing it as *empowering*. Most participants did not report any dislikes, though one mentioned, “You have to read all the time.”

**Table 4:** Acceptability themes and exemplar quotes about the *Now I Know* app

Acceptability	
Informative	“*Emphasizes preventive health”* *“Good Information”* “*It will prevent certain cervical cancers.”* “*Would become more educated of importance to completing all 3 doses.”*
Expert Advice	“*curious & wanted expert advice”* “*Answered questions I didn’t even know I had”* *“People responding [to] questions are medical professionals”*
Practical Information	*“Gave a good description & timeline of what a person needs to do.”* *“better than google, more personal”* “*A way to get firsthand knowledge quick, fast, stable.”*
Effective Reminder	*“Helpful in remembering appointment, 1st alert was 2 weeks before.”* *“I knew when the appointment was coming; I would not have remembered otherwise.”* *“Scheduling part- most useful because very busy with school.”*
Empowering	[*The app] “always has a theme that it’s never too late, & it empowers woman” “you got it girl.”* *Tells stories to lead you to make appointments.”*
Build Awareness	*“Creates awareness most people will be able to relate & not feel alone and learn.”*

Furthermore, participants provided feedback about specific features.

#### Stories

Among those who used the app, almost all stated they used the story feature the most, finding the stories relatable, helpful, empowering, and overall interesting and entertaining. “[The stories] answered questions I didn’t even know I had.” Several participants noted the stories were practical and easy to understand. They also said they were of a good length; for example, “not a medical article, easy light to read.” When asked about the major themes in the stories, many stated the stories sent a message of prioritizing preventive health; for example, “learned importance of getting HPV shots.” A few participants found the stories educated them on when to see a healthcare provider or reminded them to complete the HPV vaccine series. For example, “it was helpful to remember to make sure you get the rest of the shots.” Others noted the stories were “realistic.” When asked to discuss what they disliked about the story feature, a few participants found the stories to be too long or repetitive. For example, one participant indicated “everyone has the same line of story… from no hope to hope.” Several participants suggested adding more dialogue to make the stories more realistic.

#### Vaccine tracker/push notifications

Almost half of the participants found the vaccine tracker and appointment reminders to be helpful in reminding them about their upcoming vaccine appointments. The “scheduling part [was] most useful, being very busy with school…because it helped remember to get the vaccine,” and “I knew when the appointment was coming. I would not have remembered otherwise.” Another participant indicated they “liked schedule reminders, just right, good reminders.” However, a few participants preferred to use their own calendar for appointment reminders. Regarding the story reminder push notification, a couple of participants noted they liked them. For example, “they let me know when new stories were uploaded” or “I used the app whenever I got notifications.” However, one participant said, “I turned them off, don’t like push notifications.”

#### Question/answer (Q/A) forum

Although many read the posts, fewer than half posted questions on the Q&A forum. Some participants found the Q&A forum helpful and believed it increased awareness about topics such as routine screening for cervical cancer. “It was comprehensive,” and “it increased my awareness of HPV.” Several participants appreciated the ability to directly ask healthcare professionals questions on specific topics. One participant stated they did not have a question to post, and another was fearful their identity would be revealed. However, almost all participants who accessed the app stated it was easy to follow the Q&A on the forum.

#### Other suggested features

When asked what other features participants might like to see included in the app, some suggested a general summary or fact sheet of women’s health-related topics. One participant suggested including a game feature to make the app more interactive. Another participant suggested allowing users to customize the color scheme.

Finally, as another indication of acceptability, many indicated they *would recommend this app to others* based on its informative and helpful qualities. Some provided specific reasons such as: “I did [recommend it] to sisters/to get info, read stories, to get their vaccines;” “[because] then they get info [without] me trying to explain it and explain wrong;” “would recommend to others, good resource;” “for knowledge;” and for “people to learn and it’s interesting.” One participant was less positive about recommending the app and indicated, “I don’t know–I would mention it, but I don’t know if it offered anything new that isn’t offered on other websites. If I have questions, it’s already been asked/answered somewhere online.”

### Usability

At the 6-month exit interview, usability was assessed. Participants found the NIK app highly usable and described it as easy to navigate, well organized, and user-friendly. They appreciated its accessibility as a free app on their phones, which they always carried. The majority found the layout and tabs intuitive, allowing for quick access to specific features.

One participant stated, “downloading is easy, just have it on your phone, & notifications are easy.” The app’s visual appeal also stood out as the stories featured engaging titles and artwork that captured users’ attention. One participant valued the app’s secure environment and mentioned, “I think all women should have a secure way to know this sensitive information.” Another commented, “It empowers women, ‘you got it, girl!’” (see Table 5 for additional examples). Few participants had difficulties using the app, with the only challenges noted being “having time and remembering it’s on my phone.”

**Table 5:** Usability themes and exemplar quotes about the *Now I Know* app

Usability	
Relatable	*“From point of view from a woman.”* *“Related to stories where character is super busy”* *“Stories related to real life”* *“Most people will be able to relate.”*
Easy to read	*“Casual language-not a medical article. Easy light to read.”* *“Short & Positive”* *“Good length; can read it in between things.”*
Graphic design	*“Interesting titles and artwork”* “*Graphics were cute; liked the interface, well organized.”* *“Different tabs, clear what sections are what”*
Ease of use	*“Downloading is easy, just have on your phone & notifications are easy.”* *“Doesn’t take up too much room on the phone.” “Convenient” “Especially good with working women because it’s quick to check.”* *“It’s an app on a device you always have with you” “Almost everyone is super busy now.”* “*Go into phone & getting info [more] directly than on the web.”* *“It’s just there”* *“quick, fast, stable,* *“user-friendly”*
Privacy	*“Safer place-confide in and get solution to what to do.”* *“Yes because all women should have secure way to know this sensitive info.”* *“Free, cool, and safe place for women”*

In terms of usage, over half of the respondents reported using the NIK app since their first HPV vaccine, with most engaging with it weekly and some using it monthly. Users typically spent about 5–10 min per session, often accessing the app in the afternoon or late evening. One participant noted, “[I] browse the stories & appointments at home before I go to bed,” while another stated she checked it “mid-afternoon whenever I got a notification.” The ‘Stories’ feature was the most popular, followed by ‘Q&A’ and ‘Reminders.’

Some participants encountered barriers to regular use, such as a lack of internet access, a new phone, forgetting to use the app, or being too busy with school and work. A few noted storage limitations or technical issues as reasons for reduced usage.

### HPV vaccine completion rates

Overall, 46% completed the three-dose HPV vaccine series (Table 6). Among participants eligible for the second HPV vaccine dose (n = 35), the App group showed a higher uptake rate, with 79.2% completing the second vaccine dose, compared to 63.6% in the comparison group. For the third HPV vaccine dose, out of 31 participants, the App group again demonstrated a higher completion rate, with 55% completing the vaccine series, compared to 45.5% in the comparison group. These results indicate that participants using the App exhibited consistently higher HPV vaccine uptake at both second and third doses compared to the comparison group, suggesting the potential of the mHealth-based intervention in improving HPV vaccine series completion.

**Table 6:** HPV vaccine uptake by App group

HPV vaccine uptake	N (%)
2nd HPV vaccine uptake, n = 35	
App group	19/24(79.2%)
Comparison group	7/11(63.6%)
3rd HPV vaccine uptake, n = 31	
App group	11/20(55.0%)
Comparison group	5/11(45.5%)

## Discussion

### Summary and interpretation

The aim of the study was to assess the feasibility, acceptability, and usability of the NIK mHealth application to increase HPV vaccination completion among young, low-income minority women attending federally supported clinics. Our results demonstrated very favorable feasibility, acceptability, and usability. The NIK mHealth intervention featured stories, Q&A sessions, and reminders presented with colorful visuals in alignment with recognized best practices in health communication [38]. Participant feedback affirmed the acceptability of the app, and users were more likely to indicate that communicating with others about the HPV vaccine through the app made it easier to complete the series. Participants also noted that the app provided reliable health information in a targeted and accessible way. Such targeted interventions are often perceived as more relevant by participants, enhancing cognitive engagement and increasing the likelihood of promoting positive behavioral outcomes [39]. HPV vaccine completion content with targeted stories and reminder messages was effectively delivered to study participants. Compared to other mHealth interventions, NIK was notable in offering theory-based education that addressed salient barrier beliefs related to access, efficacy, and norms. Yet, there was still room for improvement. Based on reported reasons for limited usage, shifting the educational content to a web-based or text-based intervention may be considered. Incorporating short videos, reels, or infographics could also help minimize lengthy reading and enhance engagement. Lastly, study findings demonstrated the feasibility of implementing the NIK mHealth intervention in two different clinic settings that serve low-income young women in urban areas.

This study assessed a mHealth application intervention to improve HPV vaccination completion rates as measuring outcomes that would likely be used in future studies. It also specifically targeted young, low-income minority adult women aged 18–26 seeking care at federally supported clinics designed to improve access for those with lower socio-economic status. Study findings from our diverse sample of 35 young adult women ages 18–26 who attended publicly funded clinics and received the first dose of the HPV vaccine indicated those who received the mHealth intervention were more likely to receive the second (79% vs. 64%) and more likely to complete the third dose of the HPV vaccine (55% vs. 45%). This HPV vaccine completion rate was higher than previous mHealth intervention studies [40, 41] (among 12% who initiated the HPV vaccine). Additionally, the rates for both the intervention and comparison group were higher than national samples for vaccine completion among females in our age group (ranging from 33 to 35%) [7, 8], but closer to female college students, with rates of HPV vaccine completion of 54% [42].

### Limitation

While small sample size limited statistical power and the generalizability of the intervention on vaccine uptake outcomes, consistent positive trends detected in the data can guide future research. Recruited from an HPV vaccine initiation study, participants may have been influenced by prior involvement, potentially introducing selection bias. App usage and feedback were collected through self-reported interviews, which are subject to recall bias and social desirability bias. Participants may have over-reported favorable engagement with the intervention. This study did not address HPV vaccination costs directly, as the vaccine was covered for all study participants. Although the HPV vaccine is generally covered through most private insurance and Medicare/Medicaid, uninsured young adult women with limited financial resources may face out-of-pocket costs. By removing this potential barrier, the study may not fully capture the financial challenges that can influence vaccine completion in real-world settings.

Another limitation involves the intervention delivery method. Although NIK participants downloaded the app with study staff, ensuring they maintained the app on their phones throughout the entire study period was not possible. Some participants indicated they removed the app temporarily due to phone storage limitations, which could have reduced their exposure to the intervention content. Future studies may consider delivering HPV vaccine uptake promotion content through text messages with links, which would alleviate issues related to phone storage and app maintenance.

Finally, this study did not include young men or others who could benefit from an HPV vaccine completion intervention. Future research should encompass a broader population with content targeted to their specific contexts and risks. While the reported study findings are promising, future research with a larger and more diverse sample is needed to assess these intervention components over time.

### Conclusion and Implication

Targeted education and adherence support are crucial for achieving HPV vaccine series completion among young minority women. The NIK mHealth intervention group had higher HPV vaccine completion rates, indicating potential to address HPV vaccine disparities. Participants communicated the app’s acceptability and superiority over web searches with a more personal touch. They specifically appreciated its provision of reliable information, increased awareness, and convenient access to firsthand knowledge. Regarding usability, users found the app easy to navigate, accessible, well organized, and user-friendly. The mHealth intervention showed promise as a feasible and acceptable tool to support HPV vaccination uptake among young women. Overall, these pilot findings suggest that NIK may serve as a promising mHealth intervention for promoting HPV vaccine completion and support the need for a larger study.

## Data Availability

The datasets generated during and/or analyzed during the current study are available from the corresponding author upon reasonable request.
